# Non-invasive Early Prediction of Septic Acute Kidney Injury by Doppler-Based Renal Resistive Indexes Combined With Echocardiographic Parameters: An Experimental Study

**DOI:** 10.3389/fmed.2021.723837

**Published:** 2021-12-01

**Authors:** Ying Zhang, Jianing Zhu, Chuyue Zhang, Jing Xiao, Chao Liu, Shuo Wang, Ping Zhao, Yaqiong Zhu, Li Wang, Qiuyang Li, Yukun Luo

**Affiliations:** ^1^School of Medicine, Nankai University, Tianjin, China; ^2^Department of Ultrasound, First Medical Center, Chinese People's Liberation Army (PLA) General Hospital, Beijing, China; ^3^Medical School of Chinese People's Liberation Army (PLA), Beijing, China; ^4^Department of Nephrology, First Medical Center, Chinese People's Liberation Army (PLA) General Hospital, Beijing, China; ^5^Department of Critical Care Medicine, First Medical Center, Chinese People's Liberation Army (PLA) General Hospital, Beijing, China

**Keywords:** sepsis, acute kidney injury, renal resistive index, echocardiogram, ultrasonography

## Abstract

Non-invasive early prediction of septic acute kidney injury (S-AKI) is still urgent and challenging. Increased Doppler-based renal resistive index (RRI) has been shown to be associated with S-AKI, but its clinical use is limited, which may be explained by the complex effects of systemic circulation. Echocardiogram allows non-invasive assessment of systemic circulation, which may provide an effective supplement to RRI. To find the value of RRI combined with echocardiographic parameters in the non-invasive early prediction of S-AKI, we designed this experiment with repeated measurements of ultrasonographic parameters in the early stage of sepsis (3, 6, 12, and 24 h) in cecum ligation and puncture (CLP) rats (divided into AKI and non-AKI groups at 24 h based on serum creatinine), with sham-operated group serving as controls. Our results found that RRI alone could not effectively predict S-AKI, but when combined with echocardiographic parameters (heart rate, left ventricular end-diastolic internal diameter, and left ventricular end-systolic internal diameter), the predictive value was significantly improved, especially in the early stage of sepsis (3 h, AUC: 0.948, 95% CI 0.839–0.992, *P* < 0.001), and far earlier than the conventional renal function indicators (serum creatinine and blood urea nitrogen), which only significantly elevated at 24 h. Our method showed novel advances and potential in the early detection of S-AKI.

## Introduction

Septic acute kidney injury (S-AKI) remains one of the most important causes of acute kidney injury (AKI) in critically ill patients and is strongly associated with poor clinical outcomes. Thus, early detection is needed (1–4). At present, the diagnosis of S-AKI is still based on the changes in serum creatinine and urine output (5), but both of them can be affected by many factors and have the problem of diagnosis lag (6–8). Therefore, it is urgent to find a more sensitive method to early predict S-AKI. Moreover, the role and nature of systemic and renal hemodynamic changes remain controversial in sepsis. Due to the complex effects of inflammation, only relying on changes in renal circulation may not predict AKI (2, 9, 10).

Many methods have been proposed for the early prediction of S-AKI, especially non-invasive point-of-care ultrasound indicator—Doppler-based renal resistive index (RRI), which is given high expectations (11–14). However, most recent clinical studies have shown its limited use value, mainly because of the interference of multiple factors and substantial overlap among patients (15–17). In addition to being influenced by the vascular resistance of kidney, RRI is also influenced by systemic hemodynamics such as heart rate (HR) and cardiac output (15–17). Point-of-care echocardiogram, as a rapid and non-invasive imaging modality, allows simultaneous assessment of cardiac hemodynamics and systemic circulation (18–20), may provide an effective supplement to RRI for predicting S-AKI, and reflects the impact of inflammation to some extent. Thus, we hypothesized that the combined echocardiographic parameters and RRI could predict S-AKI. However, to the best of our knowledge, the combination of echocardiographic parameters and RRI to predict S-AKI has not been previously studied. Existing studies are either limited to using RRI (11, 14) or combined with other invasive indicators (21), but the performances of predicting S-AKI were not satisfactory.

To find the value of RRI combined with echocardiographic parameters in the non-invasive early prediction of S-AKI and minimize the interfering factors, we designed this animal experiment with repeated measurements of renal ultrasonographic and echocardiographic parameters in the early stage of sepsis (3, 6, 12, and 24 h). The aims of this study were to compare the differences in renal ultrasonographic and echocardiographic parameters between the septic AKI rats and non-AKI rats, investigating whether the combination of RRI and echocardiographic parameters can predict S-AKI.

## Materials and Methods

### Preparation of the Experimental Animals

Sprague-Dawley male rats weighing between 450 and 550 grams at the age of 12–14 weeks were supplied by the animal center of the Chinese People's Liberation Army (PLA) General Hospital (Beijing, China). All the rats were housed in a temperature-controlled room (22 ± 1°C) with a 12-h light–dark cycle and had free access to food and water. Before experiments, all the rats were fed for at least 1 week. This study was approved by the Institutional Animal Care and Use Committee of the Chinese PLA General Hospital (2019-X15-91). The experiments adhered to the Animal Research: Reporting of *In Vivo* Experiments guidelines (22). All institutional and national guidelines for the care and use of laboratory animals were followed.

During surgery and ultrasonographic measurements, animals were anesthetized by intraperitoneal injection of pentobarbital (40 mg/kg), and additional doses (8 mg/kg) were administered to maintain anesthesia when needed.

### Experimental Design and Surgical Procedure

Sepsis was induced by cecum ligation and puncture (CLP), which is among the most representative models of human peritonitis. The heterogeneity in the CLP model is similar to the clinical setting. Therefore, we established a model of relatively mild sepsis by CLP that allowed only a proportion of animals to develop into AKI within 24 h, making it possible to compare the differences between AKI and non-AKI groups.

After surgical preparation, the rats were randomly divided into two groups: CLP group and sham-operated control group. A median abdominal incision about 2 cm in length was made under sterile conditions, and the cecum was found by probing the abdominal cavity. After gently squeezing the contents in the ascending colon to fill the cecum, 25% length of cecum was ligated with a 3-0 silk suture. Two cecal leaks were formed by puncturing twice between the ligature and the cecal tip with an 18-G needle. A small amount of feces was extruded from the puncture site, and the cecum was returned to the abdominal cavity. And then the abdomen was sutured in layers. All the rats were injected with 5 ml/100 g pre-warmed normal saline (37°C) subcutaneously into the back neck immediately after the operation and were placed on a homeothermic heating pad at 37°C until waking (23). The sham-operated animals were treated identically except for subjection to cecum ligation and perforation.

**Figure 1:**
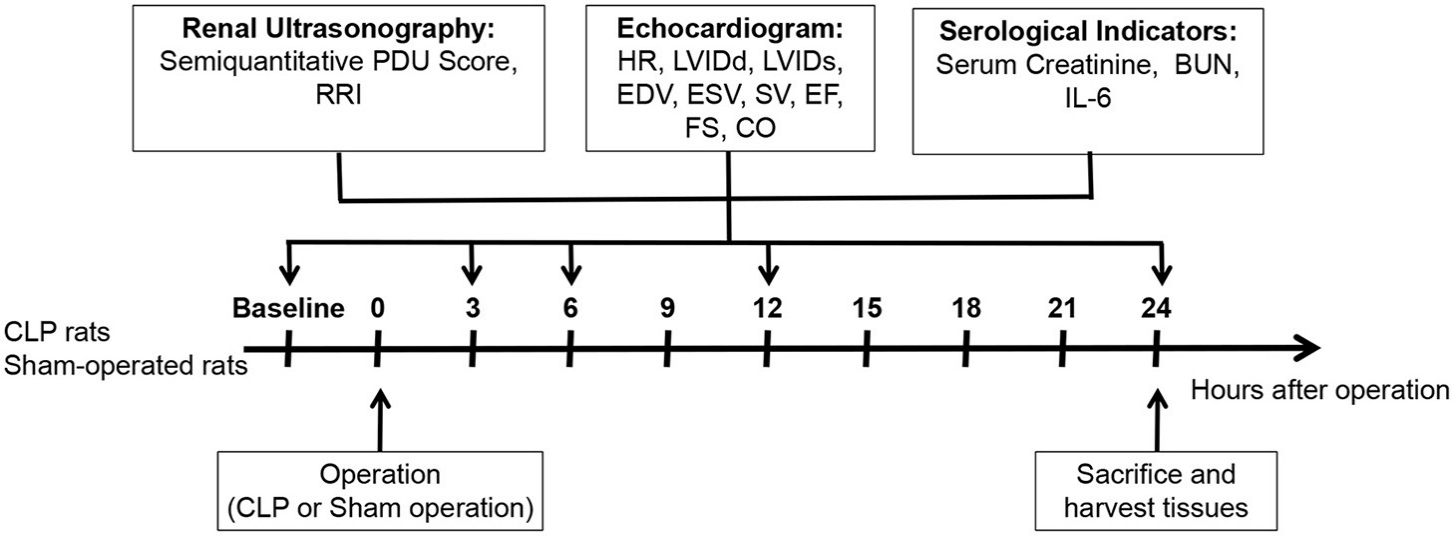
Schema of study design. Thirty-one male Sprague-Dawley rats at the age of 12–14 weeks were randomly divided into CLP group and control group. Two CLP rats were excluded due to the death after CLP, and 23 CLP rats and six sham-operated rats were finally included. Renal ultrasonography and echocardiogram were performed before and at 3, 6, 12, and 24 h after the operation, and SCr, BUN, and IL-6 at each time point were detected.

As shown in Figure 1, renal ultrasonography and echocardiogram were performed before the operation, at 3rd, 6th, 12th, and 24th h after the operation, respectively. About 0.5 ml of blood samples was collected by tail vein or retroorbital plexus puncture at each time point. After performing the renal ultrasonography and echocardiogram at 24th h after operation, the rats were sacrificed under anesthesia, and their serum and kidney samples were harvested. The CLP rats were further divided into AKI group and non-AKI group according to the 24-h serum creatinine for analysis and comparison.

### Renal Ultrasonographic and Echocardiographic Measurements

All ultrasound examinations were performed with a 7–13-MHz linear array probe (Mindray M9, Mindray, Shenzhen, China) by a radiologist with 5 years of experience in renal and cardiac ultrasonography, guaranteeing the quality of the images. And ultrasonic images were retrospectively reviewed and measured by another radiologist with 8 years of experience in renal and cardiac ultrasonography. Both radiologists were blinded to the experimental grouping. All measurements were repeated three times, and the mean values were used.

### Renal Ultrasonographic Measurements

The rats were anesthetized and placed in a supine position, and both kidneys were scanned. Renal Doppler ultrasound was performed in the kidney longitudinal scan. The Doppler gain was adjusted for maximum sensitivity at low flow without the presence of background noise, and all Doppler scans were performed at a constant gain setting for color. After identifying intra-renal vessels using color Doppler, locating an interlobar or arcuate artery, then using pulsed wave Doppler to obtain at least three consecutive similar-appearing waveforms for subsequent RRI measurements. Pulsed wave Doppler images of the arch artery or interlobar artery at three different locations (upper pole, middle, and lower pole) were retained for each kidney. Three RRI measurements were taken for each image, and the final average value was determined as the RRI of the kidney. RRI was calculated using the following formula: (peak systolic velocity − end diastolic velocity)/peak systolic velocity (11, 15).

Blood flow perfusion of the kidney was observed with power Doppler ultrasound (PDU). Optimal blood flow images were obtained and saved for offline measurement and analysis. Measurements were made at the end of expiration to avoid motion artifacts due to renal motion. Because of the higher frequency of the linear array probe, the blood flow imaging of interlobular arteries can be observed. Therefore, a modified semi-quantitative PDU scoring method was adopted for the assessment of renal blood flow perfusion in rats (Table 1) (24, 25).

**Table 1:** Modified semi-quantitative PDU score for evaluating renal perfusion.

**Grade**	**Renal perfusion**
0	Unidentifiable vessels
1	Few vessels visible in the vicinity of the hilum
1.5	Hilar and intersegmental vessels visible
2	Hilar and interlobar vessels visible in most of the renal parenchyma
2.5	Part of arcuate arteries identifiable
3	Renal vessels identifiable until the arcuate arteries in the entire field of view
3.5	Part of interlobular arteries identifiable
4	Renal vessels identifiable until the interlobular arteries in the entire field of view

*PDU, power doppler ultrasound*.

### Echocardiographic Measurements

Transthoracic echocardiography of the left ventricle was performed and analyzed on the Mindray M9 echocardiography system, as previously described. The measured hemodynamic variables included HR, left ventricular (LV) end-diastolic internal diameter (LVIDd), LV end-systolic internal diameter (LVIDs), LV end-diastolic volume (EDV), LV end-systolic volume (ESV), LV fractional shortening (FS), ejection fraction (EF), stroke volume index (SVI), and cardiac index (CI). The 7–13-MHz linear array probe was used to obtain 2D images of the parasternal long axis; M-mode images were also acquired from this position. LV long-axis and short-axis papillary muscle-level M-mode curves were recorded for measuring LV dimensions (LVIDd, LVIDs) and HR.

LV end-diastolic volume and ESV were calculated by corrected Teichholz formula: EDV = [7.0/(2.4 + LVIDd)] × LVIDd^3^; ESV = [7.0/(2.4 + LVIDs)] × LVIDs^3^. LVFS and LVEF were calculated as: LVFS = (LVIDd – LVIDs)/LVIDd × 100%; LVEF = [(EDV – ESV)/EDV] × 100%. Stroke volume (SV) and cardiac output (CO) were indexed for body weight (BW). SVI was calculated as: SVI = (EDV – ESV)/BW. CI was determined by: CI = SV × HR/BW.

### Serum Biochemical Parameters and Kidney Histology

Blood samples from each time point were centrifuged, and the serum was separated. Interleukin-6 (IL-6) was measured as a marker of systemic inflammation with an enzyme-linked immunosorbent assay (ELISA) kit following the manufacturer's instruction (IL-6 Rat ELISA Kit, Invitrogen, ThermoFisher Scientific, Carlsbad, United States). Renal function was assessed by measuring serum levels of creatinine (SCr) and blood urea nitrogen (BUN), using a biochemical analyzer (Cobas8000, Roche, Germany). AKI was defined as increasing in SCr to ≥1.5 times baseline, according to the Kidney Disease Improving Global Outcome (KDIGO) creatinine-based criteria (5).

Kidney sections (5 μm) were stained with periodic acid-Schiff (PAS) for histological analysis as previously reported (26). Two investigators independently evaluated all tissue sections under a light microscope in a blinded fashion.

### Statistical Analysis

Data are expressed as mean ± SD. Specific time point comparisons among three groups were performed using one-way ANOVA with Bonferroni's multiple comparisons test. Comparisons of repeated measurements at different time points were made using two-way repeated-measures ANOVA with the Geisser–Greenhouse correction, followed by Tukey's multiple comparisons test or Sidak's multiple comparisons test (27). Receiver-operating characteristic (ROC) curves were established for predicting AKI in a 24-h period. Binary logistic regression analysis was used to examine the association between the independent variables and the dependent variable. Taking the occurrence of AKI as the dependent variable and the renal ultrasonographic and echocardiographic measurements (RRI, HR, LVIDd, and LVIDs) as the independent variables, the logistics models at different time points (3, 6, and 12 h) were established, and ROC curves were plotted to evaluate the forecasting abilities of the logistic models. From the ROC curves, we calculated the areas under the ROC curves (AUC). DeLong's method was performed for comparison of ROC curves (28). The optimal cutoff values for sensitivity and specificity were identified from ROC curves by maximizing the Youden index (29). Since RRI is a continuous variable, and different measurements carried out on the same animal are necessarily correlated, mixed linear modeling was performed to identify the factors associated with RRI within different groups. The predefined covariates (HR, LVIDd, LVIDs, SVI, CI) were considered as fixed effects, and modeling residuals were considered as random effects. Statistical analyses were performed using GraphPad Prism v9.0 (GraphPad Software, La Jolla, United States), MedCalc v19.0.4 (MedCalc Software, Ostend, Belgium), and IBM SPSS Statistics v23.0 (IBM Corporation, Armonk, United States). Bilateral *p* ≤ 0.05 were statistically significant.

Power Analysis and Sample Size (PASS) software 15.0 (Kaysville, UT, United States) was used to estimate sample size. According to our pre-experimental results, CLP rats without AKI within 24 h were about two times as frequent as CLP rats with AKI. The calculation of the sample size was based on the areas under the ROC curves using the approach of Hanley and McNeil (30). Considering a null hypothesis at 0.50, expecting an area under the curve for the predicting AKI of 0.90 and taking into account an α-risk at 5% and a β-risk at 10%, a sample of six from the AKI group and 12 from the non-AKI group was needed. And we finally included eight rats in AKI group and 15 rats in non-AKI group.

## Results

A total of 31 rats were used in our study. Two CLP rats were excluded due to the death after CLP, and 23 CLP rats and six sham-operated rats were finally included. The CLP rats were further divided into AKI group (n = 8) and non-AKI group (*n* = 15) based on serum creatinine as previously described.

### Changes in Renal Function, Systemic Inflammation, and Renal Pathology After Sepsis

**Figure 2:**
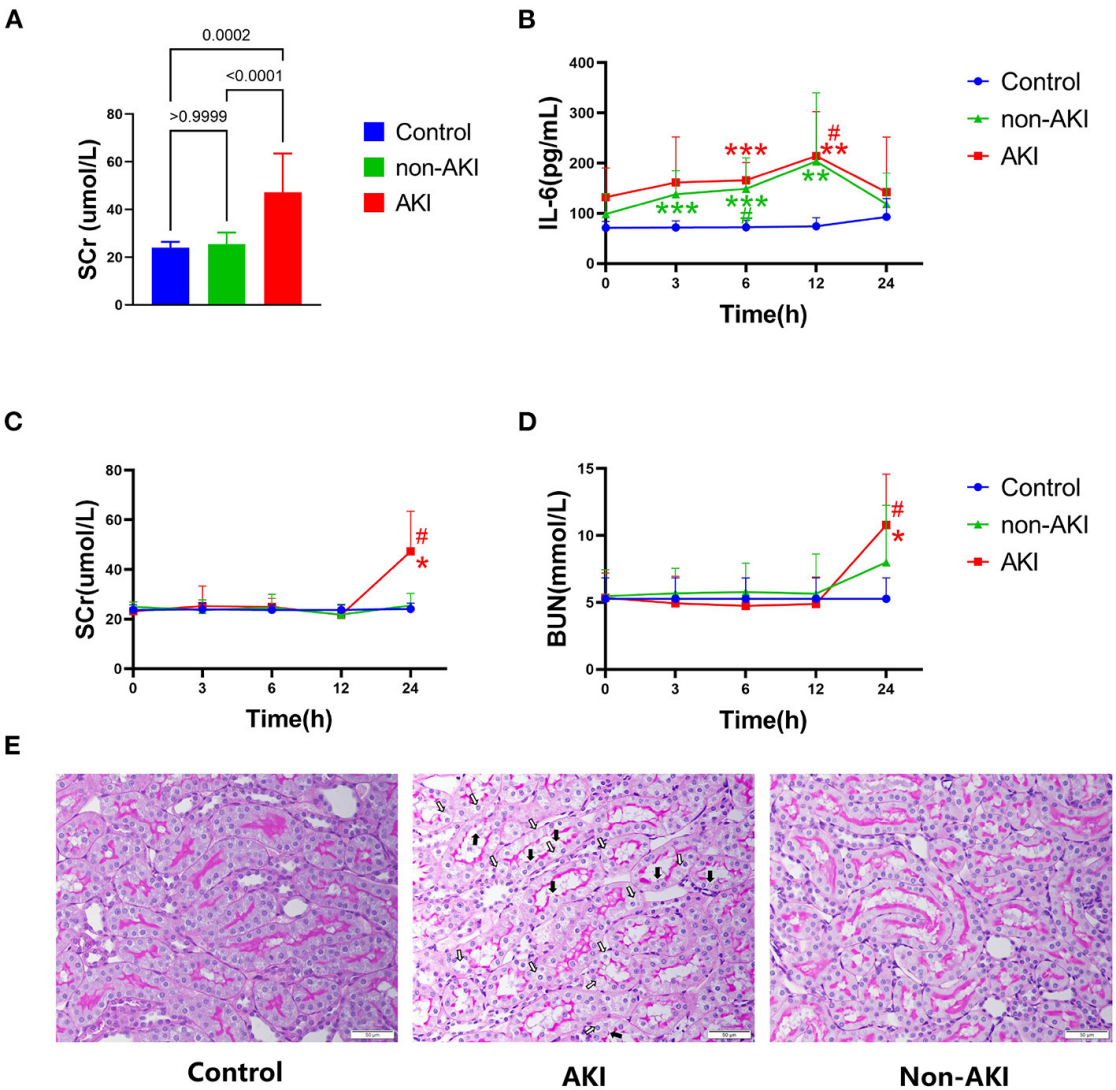
Animal groupings **(A)**, systemic inflammation index **(B)**, renal function **(C,D)**, and renal pathology **(E)** are presented. **(A)** SCr at 24 h of control, non-AKI, and AKI groups. CLP rats were further divided into the AKI group (*n* = 8) and non-AKI group (*n* = 15) based on whether the SCr at 24 h ≥ 1.5 times baseline. **(B)** Changes in IL-6 (systemic inflammation index) at baseline, 3, 6, 12, and 24 h of each group. In both AKI and non-AKI groups, the levels of IL-6 increased gradually and peaked at 12 h. **(C,D)** Significant increases in SCr and BUN were only observed at 24 h after CLP in the AKI group. (**E)** Representative microscopic images of the kidney tissues stained with periodic acid-Schiff (PAS) of control group (Left), AKI group (Middle), and non-AKI group (Right) at 24 h. Original magnification 400, bar 50 μm. In the AKI group (Middle), only minor histological changes encompassing mild brush-border loss (black arrow) and vacuolization of tubular cells (white arrow) were present on kidney histology. Data are expressed as mean ± SD. *P*-values from one-way or two-way ANOVA are shown. *P < 0.05, **P < 0.01, ***P < 0.001, compared to control group; #*P* < 0.05, compared to baseline. CLP, cecum ligation and puncture; SCr, serum creatinine; BUN, blood urea nitrogen; IL-6, interleukin-6; AKI, acute kidney injury.

Of the 23 CLP rats, eight developed S-AKI within 24 h (34.8%), which was similar to the clinical rate (8) (Figure 2A). Among eight rats that developed S-AKI, four (50%) reached KDIGO stage I, and four (50%) reached KDIGO stage II. In both AKI and non-AKI groups, the levels of IL-6 were increased gradually and peaked at 12 h, and then decreased at 24 h. No statistically significant intergroup differences were observed during the course of the experiment (Figure 2B), indicating that the inflammatory responses were similar in both groups, and the pullback of IL-6 at 24 h demonstrated the models with relatively mild inflammatory responses. However, significant increases in indexes of renal function SCr and BUN were only observed at 24 h after CLP in the AKI group (Figures 2C,D) and were not observed in non-AKI and control groups. The vacuolization of renal tubular cells and mild loss of brush border were observed in the AKI group at the end of the experiment, but no signs of acute tubular necrosis (ATN) or tubular cast formation were found (Figure 2E).

### Changes in Renal Blood Flow Detected by PDU and RRI After Sepsis

**Figure 3:**
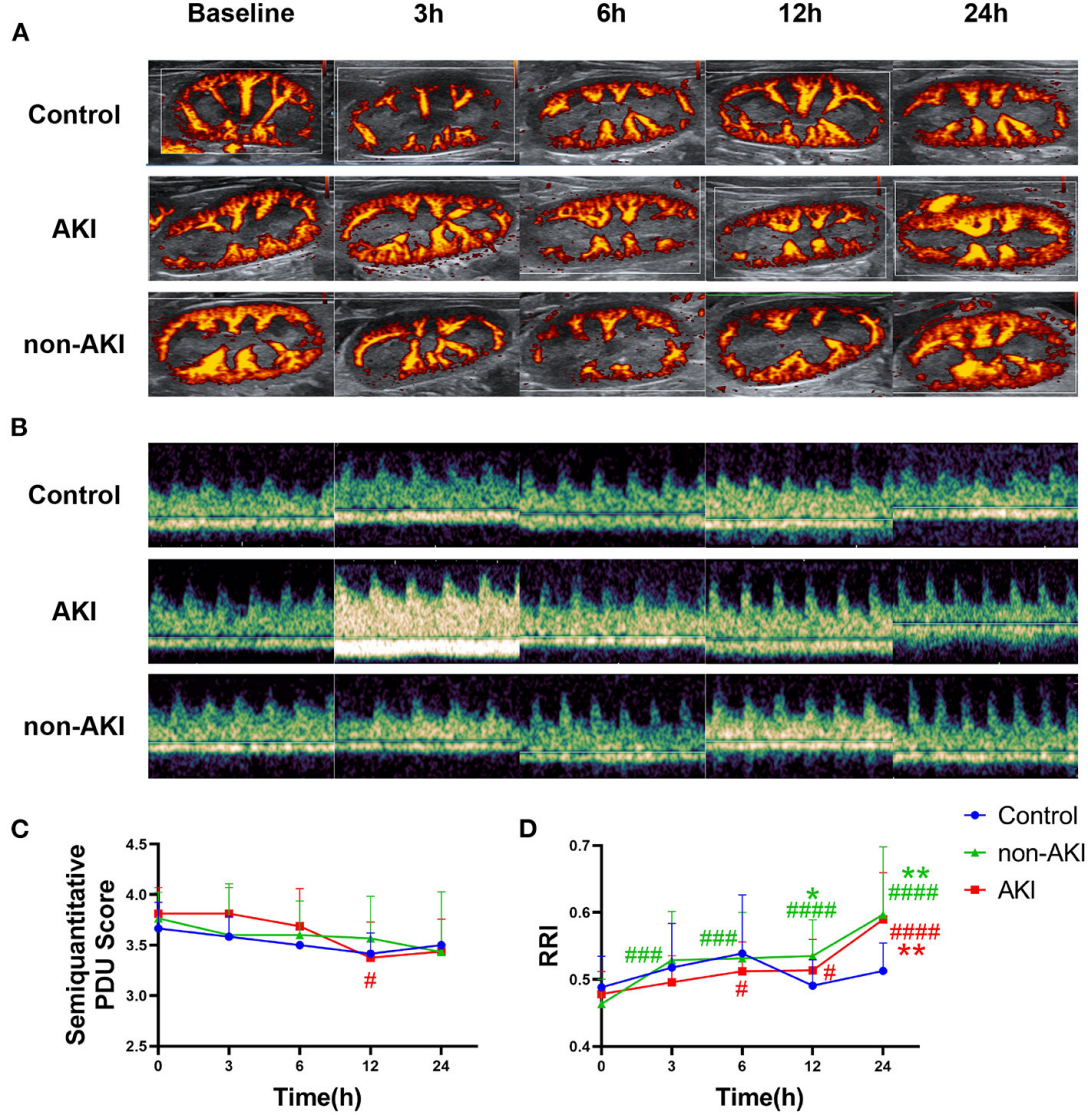
Changes in renal ultrasonography during the observation from baseline to 24 h after operation. **(A)** Example images showing the renal blood flow using power Doppler ultrasound (PDU) in control, AKI, and non-AKI groups at baseline, 3, 6, 12, and 24 h. **(B)** Example images showing the renal resistive index (RRI) using pulsed wave Doppler in control, AKI, and non-AKI groups at baseline, 3, 6, 12, and 24 h. **(C)** Changes of semi-quantitative PDU score over time. Changes in renal blood flow were relatively mild in each group, with only the AKI group showing a statistically significant decrease at 12 h from baseline. **(D)** Changes of RRI over time. RRI increased gradually in both AKI and non-AKI groups. Compared with baseline, a significant increase in RRI first appeared at 3 h in non-AKI group and at 6 h in the AKI group. Compared with the control group, the RRI of non-AKI group was significantly higher at 12 and 24 h, while significantly higher only at 24 h in the AKI group. There was no significant difference in RRI between the AKI group and the non-AKI group at each time point. Data are expressed as mean ± SD. Using two-way repeated-measures ANOVA with the Geisser–Greenhouse correction, followed by Tukey's multiple comparisons test or Sidak's multiple comparisons test, *P*-values are shown. *P < 0.05, **P < 0.01, compared to control group; #*P* < 0.05, ###*P* < 0.001, ####*P* < 0.0001, compared to baseline. AKI, acute kidney injury; PDU, power Doppler ultrasound; RRI, renal resistive index.

Changes in renal blood flow, assessed by modified semi-quantitative PDU scoring method, were relatively mild in each group (Figure 3A), with only the AKI group showing a statistically significant decrease at 12 h from baseline (3.81 ± 0.09 to 3.38 ± 0.11, *P* = 0.0353) (Figure 3C). At the 24th h, no statistical differences were observed in semi-quantitative PDU score among three groups (*P* > 0.05), although AKI had occurred. In both AKI and non-AKI groups, RRI showed an increasing trend, and no statistical differences were observed at each time point. Compared with baseline, significant increases in RRI first appeared at 3 h in non-AKI group and at 6 h in the AKI group (Figures 3B,D). Compared with the control group, the RRI of non-AKI group was significantly higher at 12 h and 24 h, while significantly higher only at 24 h in AKI group (Figure 3D). These results indicate that RRI cannot effectively predict S-AKI, and the RRI of non-AKI group increases even faster than that of the AKI group.

### Changes in Echocardiographic Parameters After Sepsis

**Figure 4:**
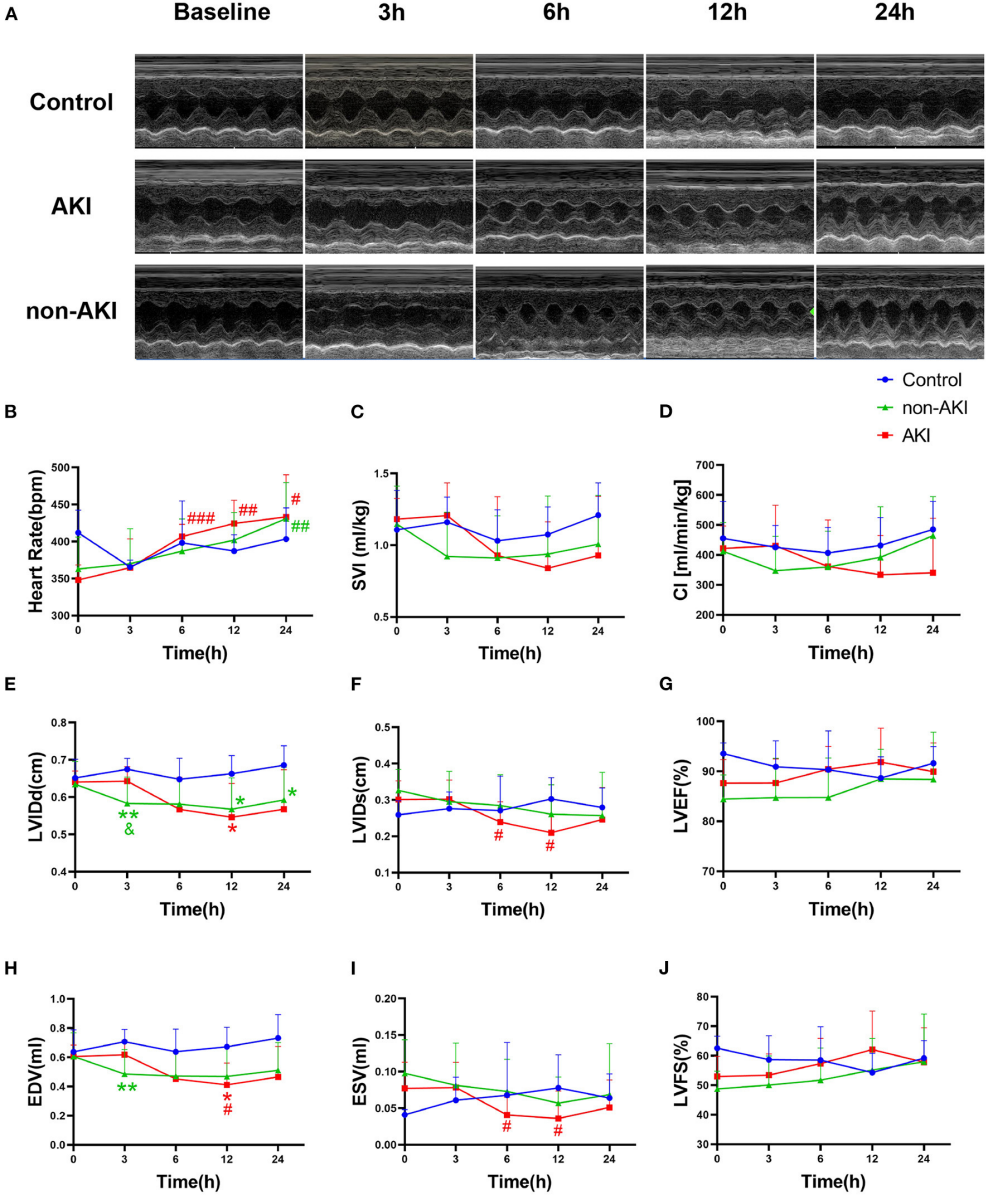
Changes in echocardiographic parameters during observation of baseline to 24 h after operation. **(A)** Example images of M-mode echocardiograms in control, AKI, and non-AKI groups at baseline, 3, 6, 12, and 24 h. **(B)** The heart rate (HR) increased gradually over time in both groups and increased faster in the AKI group than in non-AKI group. **(C,D)** The changes of stroke volume index (SVI) and cardiac index (CI) over time. In the AKI group, SVI and CI showed a decreasing trend after 3 h, whereas in the non-AKI group, they decreased at 3 h first and showed an increasing trend thereafter. **(E,H)** LVIDd and EDV showed similar changing trends with SVI. At 3 h, the LVIDd of the non-AKI group was significantly lower than that of the AKI group (*P* < 0.05). **(F,I)** LVIDs and ESV of AKI group were significantly lower than those of the baseline at 6 h and 12 h (*P* < 0.05). **(G,J)** The LVEF and LVFS during observation were not statistically different in each group. Data are expressed as mean ± SD. Using two-way repeated-measures ANOVA with the Geisser–Greenhouse correction, followed by Tukey's multiple comparisons test or Sidak's multiple comparisons test, *P*-values are shown. **P < 0.01, compared to control group; #*P* < 0.05, ##*P* < 0.01, ###*P* < 0.001, compared to baseline. &*P* < 0.05, compared to the AKI group. AKI, acute kidney injury; SVI, stroke volume index, stroke volume indexed by body weight; CI, cardiac index, cardiac output indexed by body weight; LVIDd, left ventricular end-diastolic internal diameter; LVIDs, left ventricular end-systolic internal diameter; EDV, left ventricular end diastolic volume; ESV, left ventricular end systolic volume; SV, stroke volume; LVEF, left ventricular ejection fraction; LVFS, left ventricular fractional shortening.

The HR increased gradually over time in both AKI and non-AKI groups, and no statistical differences were observed between the two groups (Figures 4A,B). The upward trend of HR was more pronounced in the AKI group than in non-AKI group, and HR was significantly higher than the baseline at 6 h in the AKI group (348.13 ± 7.15 to 406.67 ± 5.88 bpm, *P* = 0.0007), while at 24 h in non-AKI group (362.84 ± 11.16 to 430.93 ± 12.58 bpm, *P* = 0.0036) (Figure 4B). Although the changes in SVI and CI over time were not statistically different, they showed similar trends. That is, in the AKI group, SVI and CI did not change significantly or slightly increased at 3 h, gradually decreased thereafter, to a minimum at 12 h, and rebounded at 24 h. The difference was that in the non-AKI group, SVI and CI first decreased at 3 h and then increased gradually and slowly (Figures 4C,D). The changing trends described above could also be observed in LVIDd and EDV. At 3 h, the LVIDd of the non-AKI group was significantly lower than that of the AKI group (0.58 ± 0.02 to 0.64 ± 0.01 cm, *P* = 0.0417) (Figures 4E,H). LVIDs and ESV of the AKI group were significantly lower than those of the baseline at 6 h (LVIDs: 0.30 ± 0.02 to 0.24 ± 0.02 cm, *P* = 0.0455; ESV: 0.08 ± 0.01 to 0.04 ± 0.01 ml, *P* = 0.0286) and 12 h (LVIDs: 0.30 ± 0.02 to 0.21 ± 0.03 cm, *P* = 0.0212; ESV: 0.08 ± 0.01 to 0.04 ± 0.01 ml, *P* = 0.0346) (Figures 4F,I). The LVEF and LVFS showed a slow upward trend in AKI and non-AKI groups, but there was no statistical difference between and within groups (Figures 4G,J). The above results indicated that there were differences in systemic hemodynamic changes between AKI group and non-AKI group, and the differences could be observed in the very early stage (3 h), which may predict the occurrence of AKI.

### Predictive Value for S-AKI of RRI Combined With Echocardiographic Parameters

In order to evaluate the predictive value of RRI combined with echocardiography for AKI, logistic regression models were established to predict AKI. LVIDd and LVIDs are direct measurement indicators, and other related indicators are calculated by them. Therefore, LVIDd and LVIDs were mainly included, together with HR and RRI; logistic regression analyses were done separately at each time point.

**Figure 5:**
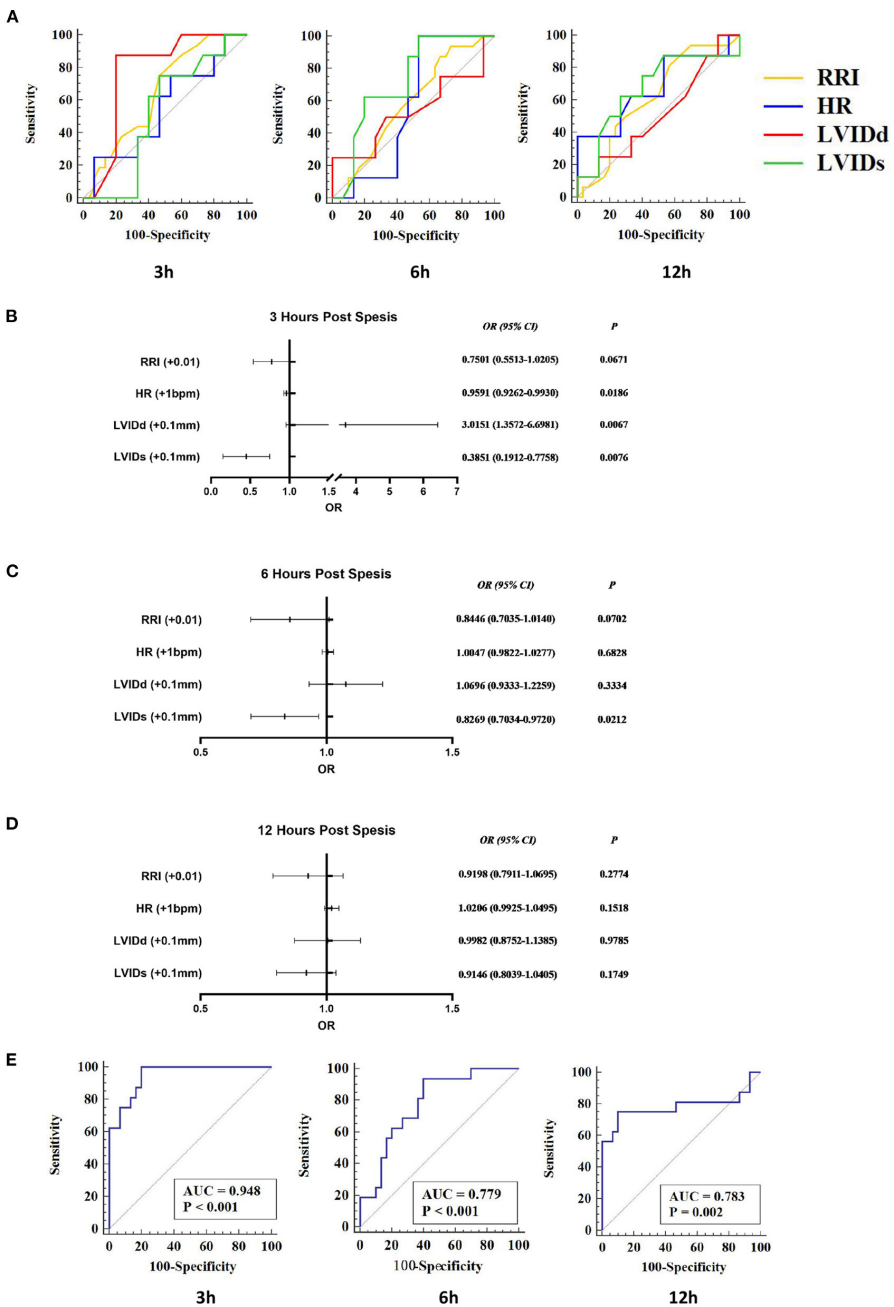
RRI combined with echocardiographic parameters to predict S-AKI. RI, HR, LVIDd, and LVIDs were included in the logistics regression analysis to explore the joint predictive value of S-AKI. **(A)** Receiver-operating characteristic (ROC) curve of RRI, HR, LVIDd, and LVIDs at 3 h (Left), 6 h (Middle), and 12 h (Right). At 3 h, only LVIDd had a predictive value for AKI (AUC = 0.771, 95% CI 0.623–0.882, *P* < 0.001). At 6 h, only LVIDs had a predictive value for AKI (AUC = 0.725, 95% CI 0.574–0.846, *P* = 0.003). At 12 h, only HR was predictive of AKI (AUC = 0.679, 95% CI 0.525–0.809, *P* = 0.047). **(B–D)** shows the results of logistic regression analysis of RI, HR, LVIDd, and LVIDs at 3 h **(C)**, 6 h **(D)**, and 12 h **(E)** of S-AKI. At 3 h **(B)**, animals with decreased HR (OR = 0.9591) and LVIDs (OR = 0.3851) and increased LVIDd (OR = 3.0151) were more likely to develop AKI. **(E)** ROC curve of the logistic regression models of 3 h (Left), 6 h (Middle), and 12 h (Right). The area under ROC curves of the 3, 6, and 12-h logistic regression models were 0.948 (95% CI 0.839–0.992, *P* < 0.001), 0.779 (95% CI 0.633–0.888, *P* < 0.001), and 0.783 (95% CI 0.637–0.891, *P* = 0.002), respectively. RRI, renal resistive index; S-AKI, septic acute kidney injury; HR, heart rate; LVIDd, left ventricular end-diastolic internal diameter; LVIDs, left ventricular end-systolic internal diameter.

At 3 h, only LVIDd had a predictive value for AKI (AUC = 0.771, 95% CI 0.623–0.882, *P* < 0.001). At 6 h, only LVIDs had a predictive value for AKI (AUC = 0.725, 95% CI 0.574–0.846, *P* = 0.003). At 12 h, only HR was predictive for AKI (AUC = 0.679, 95% CI 0.525–0.809, *P* = 0.047) (Figure 5A). Logistic regression analyses showed that RRI was not an independent factor of AKI (*p* > 0.05). At 3 h, animals with decreased HR (OR = 0.9591) and LVIDs (OR = 0.3851) and increased LVIDd (OR = 3.0151) were more likely to develop AKI. At 6 h, only LVID is an independent factor, and AKI was more likely to occur when it declined (OR = 0.8269). At 12 h, although the logistic regression model had a predictive effect, no indicator was an independent influencing factor for the occurrence of AKI (Figures 5B–D).

While combining RRI, HR, LVIDd, and LVIDs to establish binary logistic regression models, the prediction performance of the models was significantly improved (Figure 5E). The area under the ROC curve of the 3, 6, and 12-h logistic regression models was 0.948 (*P* < 0.001), 0.779 (*P* < 0.001), and 0.783 (*P* = 0.002), respectively. Detailed results from logistic regression models for the combination of RRI and echocardiography parameters are presented in Table 2 and Figure 5.

**Table 2:** Binary logistic regression model for predicting S-AKI.

**Variable-3 h**	**Coefficient**	**Std. error**	**Wald**	***P***	**Odds ratio**	**95% CI**
RRI (+0.01)	−0.2876	0.1571	3.3520	0.0671	0.7501	0.5513–1.0205
HR (+1 bpm)	−0.0418	0.01777	5.5362	**0.0186**	0.9591	0.9262–0.9930
LVIDd (+0.1 mm)	1.1036	0.4072	7.3445	**0.0067**	3.0151	1.3572–6.6981
LVIDs (+0.1 mm)	−0.9542	0.3573	7.1320	**0.0076**	0.3851	0.1912–0.7758
Constant	−10.7397	11.8310	0.8240	0.3640		
**Variable-6 h**	**Coefficient**	**Std. error**	**Wald**	***P***	**Odds ratio**	**95% CI**
RRI (+0.01)	−0.1689	0.0933	3.2782	0.0702	0.8446	0.7035–1.0140
HR (+1 bpm)	0.0047	0.0116	0.1670	0.6828	1.0047	0.9822–1.0277
LVIDd (+0.1 mm)	0.0673	0.06959	0.9358	0.3334	1.0696	0.9333–1.2259
LVIDs (+0.1 mm)	−0.1901	0.0825	5.3080	**0.0212**	0.8269	0.7034–0.9720
Constant	7.3733	9.2934	0.6295	0.4275	0.8446	0.7035–1.0140
**Variable-12 h**	**Coefficient**	**Std. error**	**Wald**	***P***	**Odds ratio**	**95% CI**
RRI (+0.01)	−0.0836	0.0769	1.1796	0.2774	0.9198	0.7911–1.0695
HR (+1 bpm)	0.0204	0.0142	2.0537	0.1518	1.0206	0.9925–1.0495
LVIDd (+0.1 mm)	−0.0018	0.0671	0.0007	0.9785	0.9982	0.8752–1.1385
LVIDs (+0.1 mm)	−0.0893	0.0658	1.8407	0.1749	0.9146	0.8039–1.0405
Constant	−2.5140	8.0051	0.0986	0.7535	0.9198	0.7911–1.0695

*S-AKI, septic acute kidney injury; RRI, renal resistive index; HR, heart rate; LVIDd, left ventricular end-diastolic internal diameter; LVIDs, left ventricular end-systolic internal diameter; Std. Error, Standard error; CI, Confidence interval. Statistically significant values (P < 0.05) are shown in bold*.

### Influencing Factors of RRI

In order to clarify whether there are differences in the effects of systemic circulation on RRI among different groups, linear mixed models for RRI were established for each group. HR, LVIDd, LVIDs, SVI, and CI were tested for relevance with RRI (Table 3). The decrease in LVIDs and SVI was associated with the increase of RRI in the control and non-AKI groups. The increase in HR and SVI was associated with the increase of RRI in the AKI group, which was contrary to non-AKI group. And the CI decrease was associated with the RRI increase in the AKI group.

**Table 3:** Linear mixed model for predicting renal resistive index (RRI).

**RRI from baseline to 24 h in Control group**	**Coefficient**	**95% CI**	***P***
**Fixed effects**			
HR (+1 bpm)	−0.0003	−0.0006; 0.0013	0.492
LVIDd (+0.1 mm)	0.0384	0.0243; 0.0526	**<0.001**
LVIDs (+0.1 mm)	−0.0023	−0.0057; 0.0010	**<0.001**
SVI (+1 ml/kg)	−0.4726	−0.8331; −0.1122	**0.011**
CI (+1 ml/min/kg)	−0.0006	−0.0013; 0.0000	0.058
**RRI from 3 to 24 h after CLP in non-AKI group**	**Coefficient**	**95% CI**	***P***
**Fixed effects**			
HR (+1 bpm)	−0.0010	−0.0019; −0.0001	**0.031**
LVIDd (+0.1 mm)	0.0063	−0.0008; 0.0134	0.083
LVIDs (+0.1 mm)	−0.0052	−0.0084; 0.0021	**0.001**
SVI (+1 ml/kg)	−0.4116	−0.7824; −0.0408	**0.030**
CI (+1 ml/min/kg)	0.0009	−0.0001; 0.0018	0.068
**RRI from 3 to 24 h after CLP in AKI group**	**Coefficient**	**95% CI**	***P***
**Fixed effects**			
HR (+1 bpm)	0.0020	0.0007; 0.0033	**0.003**
LVIDd (+0.1 mm)	−0.0034	−0.0128; 0.0060	0.471
LVIDs (+0.1 mm)	0.0038	−0.0002; 0.0077	0.060
SVI (+1 ml/kg)	0.5373	0.0781; 0.9965	**0.023**
CI (+1 ml/min/kg)	−0.0012	−0.0023; −0.0001	**0.032**

*All the continuous covariates, the coefficient associated with the considered covariate corresponds to the impact on renal resistive index of the increase of one unit in this covariate*.

*RRI, renal resistive index; AKI, acute kidney injury; HR, heart rate; LVIDd, left ventricular end-diastolic internal diameter; LVIDs, left ventricular end-systolic internal diameter; SVI, stroke volume index; CI, cardiac index. Statistically significant values (P < 0.05) are shown in bold*.

## Discussion

In this study, we mainly explored whether non-invasive renal ultrasonographic and echocardiographic parameters could predict S-AKI at early stage and found that non-invasive ultrasonographic parameters could change prior to the conventional renal function indicators SCr and BUN, thus further predicting S-AKI. Among all the ultrasonographic parameters in preclinical and clinical studies, RRI has received the most attention but did not show an individual predictive value in this study. However, there were differences in echocardiographic parameters, which represent the systemic circulation, between the AKI group and non-AKI group, and the differences could be observed in the very early stage (3 h). These suggested that animals developing S-AKI experienced a delayed (after 3 h) and more obvious decline in macro-circulatory parameters, which eventually led to AKI. Furthermore, combining RRI with echocardiographic parameters at each time point showed good predictive values for S-AKI, especially at the early stage (3 h) after CLP, with the AUC of 0.948. Finally, we analyzed the effects of indexes on RRI among different groups and found that the trends of the effects of macro-circulatory parameters on RRI were opposite in the AKI and non-AKI groups. Our method showed novel advances and potential in the early detection of S-AKI.

In order to explore the potential early prediction method of S-AKI, four time points (3, 6, 12, and 24 h) were selected, and relatively mild sepsis models by CLP were established. Therefore, only about one-third of animals developed AKI within 24 h, which was similar to the clinical rate (8). In addition, the renal pathology at 24 h showed relatively mild pathological damage, and no signs of ATN or tubular cast formation were found. This is consistent with the results from other studies, demonstrating that the degree of pathological changes in S-AKI, which were relatively mild, is not proportional to functional impairment as determined by SCr (31–33). Therefore, the conclusions drawn from this study may be more in line with the clinical situation.

In this study, RRI and semi-quantitative PDU scores cannot predict S-AKI alone, indicating that the hemodynamic changes of the kidney cannot explain all the causes of S-AKI. This phenomenon is in agreement with a recent clinical study by Zhi et al. (11), showing that both RRI and semi-quantitative PDU scores performed poorly in predicting AKI in patients with sepsis, while valuable in patients with heart failure. Studies by Beloncle et al. and Lerolle et al. (13, 14) also showed that in septic AKI and non-AKI patients, RRI had a large overlap, which limited its clinical application. Previous clinical studies in which RRI performed well in predicting AKI mostly focused on AKI caused by renal ischemia (after major surgery or heart failure) (34–36). However, in clinical studies that did not distinguish AKI subtypes, RRI performed relatively poor, as well as semi-quantitative PDU scores (24). This may be explained by the fact that the mechanism of S-AKI, which accounts for a major portion of AKI occurrence in critically ill patients, is different from AKI that caused by renal ischemia (2, 37, 38). Relevant studies have also shown that S-AKI may develop in the absence of renal hypoperfusion and clinical signs of hemodynamic instability, and in the presence of normal or increased global renal blood flow (8). Therefore, our study combined renal hemodynamic indexes and systemic hemodynamic indexes to see whether S-AKI can be predicted.

Our results showed that echocardiographic parameters, which represented systemic hemodynamic changes, may be early sensitive indicators of S-AKI. In the present study, the echocardiographic parameters of AKI group had more obvious changes than those of non-AKI group, which was characterized by the delayed (after 3 h) decline. This may be explained by the fact that the AKI group experienced systemic hemodynamic changes from hyperdynamic to hypodynamic after CLP (39–41), combined with inflammatory effects and alterations in renal circulation, which in turn caused more severe damage to the kidney. Therefore, we combined RRI with the most directly measured echocardiographic parameters (HR, LVIDd, LVIDs), to find the non-invasive and simple way to predict S-AKI. Encouragingly, the combination in the very early stage of sepsis (3 h) showed impressive specificity and sensitivity (AUC: 0.948). This may be explained by the different renal and systemic hemodynamic change trends at the beginning, which eventually led to different outcomes of the kidney. To our knowledge, early non-invasive prediction of S-AKI at this time point (3 h after sepsis) has not been reported. This finding can also partially explain the clinically confusing and contradictory results, as a large proportion of critically ill patients have already entered the middle to late phase of sepsis at the time of admission, and the differences in ultrasonographic parameters have been reduced.

And the combination of RRI, HR, LVIDd, and LVIDs may actually represent different hemodynamic alterations in response to sepsis: RRI represented the resistance and compliance of the renal vasculature (16); HR represented the myocardial oxygen demand (42); LVIDd represented the cardiac preload (43); LVIDs represented the cardiac afterload (43). The results of logistics regression analysis at 3 h after sepsis showed that increased HR (OR = 0.9591, *P* = 0.0186) and LVIDs (OR = 0.3851, *P* = 0.0076) were protective factors, and increased LVIDd (OR = 3.0151, *P* = 0.0067) was a risk factor for developing AKI. These results were actually concordant with the previously published studies (2, 32), for increased LVIDd reflecting elevated preload and venous reflux and may indirectly reflect elevated central venous pressure, which was considered being associated with AKI (44). Simultaneously, increased HR and LVIDs indirectly reflected that the effective circulating blood volume was relatively maintained and vasoplegia was relatively mild. At 3 h, the RRI of the non-AKI group increased more significantly than that of the AKI group, which may be explained by the more severe vasoplegia in the AKI group, leading to a relatively unchanged or decreased RRI (45).

The factors affected by RRI among different groups were also explored in this study. Interestingly, although the RRI increased in both groups, the trends of RRI corresponding to the parameters of systemic circulation were quite different. Previous studies have shown that RRI is negatively correlated with HR in normal populations, whereas it is positively correlated with central pulse pressure and cardiac output [15–17, 46, 47]. In this study, the RRI changes in the non-AKI group still conformed to this trend, while the AKI group showed the opposite, indicating that the regulation of renal circulation in sepsis with AKI is different from that without AKI (12), and also confirming the need to combine RRI with echocardiographic parameters.

Our study also has some limitations. First, to find a completely non-invasive method to predict S-AKI and minimize the effects caused by invasive procedures, no invasive procedures have been implemented to measure indicators such as mean arterial pressure, which may be complementary. Besides, since limited samples were collected, SCr, BUN, and IL-6 were mainly selected for testing. Finally, the sepsis in this study was relatively mild, and the value of RRI combined with echocardiographic parameters in predicting S-AKI in moderate-to-severe sepsis may require further study.

## Conclusions

Our results found that RRI alone could not effectively predict S-AKI, but when combined with echocardiographic parameters (HR, LVIDd, LVIDs), the predictive value had been significantly improved, especially in the early stage of sepsis (3 h), and far earlier than the conventional renal function indicators (SCr and BUN), which only significantly elevated at 24 h. We hope that these findings can be helpful to the clinic and form the basis for further research and application in the clinic.

## Data Availability Statement

The original contributions presented in the study are included in the article, further inquiries can be directed to the corresponding authors.

## Ethics Statement

The animal study was reviewed and approved by the Institutional Animal Care and Use Committee of the Chinese PLA General Hospital.

## Author Contributions

YZha, JZ, CZ, JX, CL, and YL designed the research. YZha, JZ, CZ, JX, CL, SW, PZ, and LW performed the research. YZha, JZ, CZ, JX, YZhu, LW, QL, and YL methodology. YZha, JZ, CZ, and CL analyzed the data. YZha, JZ, CZ, YZhu, QL, and YL wrote the manuscript. All authors revised the manuscript draft and approved the final version for submission.

## Funding

This study was supported by the National Natural Science Foundation of China (81971635, 81801698, and 82001817), New Technology Cultivation Support Fund of Chinese PLA General Hospital (XJS-202105), Construction Projects of Key Military Academies and Key Disciplines in the 13th Five-Year Plan (A350109), and Chinese Postdoctoral Science Foundation (2020T130778).

## Conflict of Interest

The authors declare that the research was conducted in the absence of any commercial or financial relationships that could be construed as a potential conflict of interest.

## Publisher's Note

All claims expressed in this article are solely those of the authors and do not necessarily represent those of their affiliated organizations, or those of the publisher, the editors and the reviewers. Any product that may be evaluated in this article, or claim that may be made by its manufacturer, is not guaranteed or endorsed by the publisher.
